# Transcriptome analyses revealed molecular responses of *Cynanchum auriculatum* leaves to saline stress

**DOI:** 10.1038/s41598-019-57219-8

**Published:** 2020-01-16

**Authors:** Ming Zhang, Li-Zhou Hong, Min-Feng Gu, Cheng-Dong Wu, Gen Zhang

**Affiliations:** 1 Xinyang Agricultural Experiment Station of Yancheng City, Jiangsu Province, 224045 P.R. China; 2 Shenzhen GenProMetab Biotechnology Company Limited., Shenzhen, Guangdong Province 51800 P.R. China

**Keywords:** RNA sequencing, Physiology

## Abstract

*Cynanchum auriculatum* is a traditional herbal medicine in China and can grow in saline soils. However, little is known in relation to the underlying molecular mechanisms. In the present study, *C. auriculatum* seedlings were exposed to 3.75‰ and 7.5‰ salinity. Next, transcriptome profiles of leaves were compared. Transcriptome sequencing showed 35,593 and 58,046 differentially expressed genes (DEGs) in treatments with 3.75‰ and 7.5‰, compared with the control, respectively. Kyoto Encyclopedia of Genes and Genomes (KEGG) analyses of these DEGs enriched various defense-related biological pathways, including ROS scavenging, ion transportation, lipid metabolism and plant hormone signaling. Further analyses suggested that *C. auriculatum* up-regulated Na^+^/H^+^ exchanger and V-type proton ATPase to avoid accumulation of Na^+^. The flavonoid and phenylpropanoids biosynthesis pathways were activated, which might increase antioxidant capacity in response to saline stress. The auxin and ethylene signaling pathways were upregulated in response to saline treatments, both of which are important plant hormones. Overall, these results raised new insights to further investigate molecular mechanisms underlying resistance of *C. auriculatum* to saline stress.

## Introduction

Tuberous root of *Cynanchum auriculatum* is a traditional herbal medicine in Asian countries. It has been listed in Chinese Pharmacopoeia and Korean Pharmacopoeia, namely “Baishouwu” and “Baekshuoh”, respectively^1^. Pharmacological researches revealed that application of *C. auriculatum* could enhance immunity^2^, display activities of anti-tumor^3^, antioxidant^4^ and gastro protection^2^. Thus, *C. auriculatum* has high economic values.

In China, *C. auriculatum* is cultivated in Shandong, Jiangsu, and Anhui provinces. Approximately 95% of *C. auriculatum* are produced at Binhai County, Yancheng City (China), which is a coastal city. Due to human activities and natural seawater erosion, most agricultural lands there show severe salinization. Cultivation on saline soils and/or using brackish water resources has attracted widespread attentions in recent years. Planting *C. auriculatum* on saline soils may be an alternative approach to solve the problem of insufficient agricultural lands, thus increasing the total production and decreasing the unit price of *C. auriculatum*. However, there are no reports investigating responses of *C. auriculatum* to saline stress.

The genus *Cynanchum* (Linn.) includes approximately 200 species in the world, which are widely distributed in eastern Africa, Mediterranean region, tropical, subtropical and temperate regions of Eurasia^5^. There are 53 species and 12 varieties of this genus in China, which are mainly distributed in southwest provinces^6^. The complete chloroplast genome sequence has been sequenced for *C. auriculatum* and *C. wilfordii*, and phylogenetic analysis revealed that these two species are evolutionarily close^7,8^. According to previous studies, plants in *Cynanchum* showed strong tolerance to salinity. It was found that the seed germination and radicle length of swallowwort (*Cynanchum acutum* L.) decreased with increased saline stress, which is more tolerant to salinity than common milkweed, hairy beggarticks, and scotch thistle^9^. As a halophyte speices, *Cynanchum chinense* increases the content of osmotic regulators such as soluble sugar, betaine and organic acids in saline-alkali environments, and its osmotic regulators content is higher than plants in *Poaceae*, *Leguminosae*, *Cyperaceae* and *Boraginaceae* families^10^. *C. auriculatum* is closely related to *C. acutum* and *C. chinense*^11^, and may also have similar tolerance to saline stress.

Besides antioxidant regulation, plants have also evolved other physiological and molecular mechanisms to resist saline stress^12^. For example, plant hormones, such as abscisic acid (ABA), auxin and ethylene, could function as essential endogenous signal molecules and regulate plant development and tolerance to salinity^13,14^. The WRKY and MYB transcription factors are important to the regulation of transcriptional reprogramming involved in abiotic stress responses^15,16^, which have been demonstrated to regulate the contents of chlorophyll, proline, soluble sugar as well as activities of superoxide dismutase (SOD), and catalase (CAT) under saline stress^17,18^. In response to saline stress, metabolism of unsaturated fatty acid was activated to produce various compounds, which then function as signal molecules or participate in plant defense system as antimicrobial substances^19–21^. Whether these mechanisms also function in *C. auriculatum* resistance to salinity should be investigated.

Transcriptome sequencing is an ideal method to investigate molecular changes in response to saline stress in plants^22–24^. In the present study, to investigate molecular changes of *C. auriculatum* to saline environments, seedlings were exposed to saline treatments. Next, leaves were subjected to transcriptome sequencing, and bioinformatics analyses were conducted to globally predict effects of salinity on physiology of *C. auriculatum*. These results might contribute theoretical basis to guide *C. auriculatum* cultivation on saline soils.

## Materials and Methods

### Ethics statement

No specific permit is required for the present study in P. R. China.

### Cultivation of *C. auriculatum*

Seeds of *C. auriculatum* were collected from the planting base at Xinyang Agricultural Experiment Station of Yancheng City (Yancheng, P. R. China) on 2017. Full and healthy *C. auriculatum* seeds were washed with sterile water for 2–3 times, soaked in 2% NaClO solution for 2 min, and then washed with sterile water for 3 times. After germination in sterile water, the seedlings were cultured in 1/2 Hoagland’s solution at 25 °C under a photoperiod of 16 h:8 h (light:dark). The light intensity was 14,400 lux.

### Treatments with salinity

Based on the preliminary results, saline treatments included two salinities, 3.75‰ and 7.5‰ (dissolving commercial sea salts in 1/2 Hoagland’s solution). The experiments were carried out in plastic boxes (20 cm * 10 cm * 8 cm). In each box, 1 L of solution was added. For saline treatments, suitable amount of sea salts were added in 1/2 Hoagland’s solution to achieve salinities of 3.75‰ and 7.5‰. 1/2 Hoagland’s solution without extra addition of sea salts was also prepared as the control (CT). Each treatment consisted of five individuals and repeated three times independently. To avoid effects of evaporation on salinity, the culture media were renewed every three days. After 15 days, the top four leaves from five individuals in each treatment were collected and mixed as one sample. The plants were taken out, and quickly dried using sterile filter paper. After freezing with liquid nitrogen, leaves were stored at −80 °C until RNA extraction.

### Transcriptome sequencing

The Biozol reagent produced by Bioer Technology (Hangzhou, China) was used to extract total RNA according to the manufacture’s protocol. The integrity of RNA was visualized by 1% agarose gel electrophoresis, and its concentration and purity were detected using a NanoDrop Microvolume Spectrophotometers and Fluorometer (ThermoFisher, Shanghai, China). Oligo(dT) magnetic beads (NEB, USA) were used to enrich mRNA in the samples and then sequencing libraries were prepared using NEBNext mRNA Library Prep Master Mix Set for Illumina (NEB, USA) as described in the manufacture’s protocol. AMPure XP beads (Beckman, Germany) were used to purify the DNA fragments with 250–300 bp length, which were further amplified by PCR using Phusion High-Fidelity DNA polymerase. Qubit 2.0 software was used for preliminary quantitative analysis of the libraries. The library was diluted to 1.5 ng/μL to check the distribution of insert size using an Agilent Bioanalyzer 2100 system. Real-time quantitative PCR (RT-qPCR) was applied to determine the accurate concentration of library (concentration > 2 nmol was the effective concentration of the library). Finally, Illumina HiSeq. 4000 platform was used to sequence the DNA libraries.

### Bioinformatics analyses

To determine whether sequencing data were suitable for subsequent analyses, quality control (QC) of raw reads was performed first. Reads with low quality (with >20% bases having Phred quality score ≤10), joint contamination and/or high ratio of unknown base (ratio ≥5%) were removed. Clean reads were assembled using the Trinity program^25^, and the longest transcript of each sequence was selected for the follow-up analysis of unigene. After assembly, the gene expression levels were compared between treatments and the control by calculating FPKM (expected number of fragments per kilobase of transcript sequence per million base pairs sequenced) values of each unigene using RSEM v1.2.8^26^. Genes with fold change ≥ 2 and Q-value (adjusted P-value) ≤ 0.001 were considered as significantly differentially expressed genes (DEGs).

TransDecoder software was used to identify candidate coding sequence (CDS). The longest open reading frame was extracted for each unigene, and then the Pfam protein homologous sequences were searched by blasting against SwissProt and Hmmscan database to predict CDS^27^. For gene ontology (GO) annotations, the DEGs were blasted against the GO database using Blast2Go program (http://www.blast2go.com/Ver.2.3.5)^28^. For Kyoto Encyclopedia of Genes and Genomics (KEGG) annotations, all the DEGs were mapped to the KEGG database (https://www.genome.jp/kegg/pathway.html) using BLASTX^29^. Comparisons with Q-value ≤ 0.05 were considered statistically significant.

### Real-time quantitative PCR

To verify the reliability of RNA-seq results, nine unigenes were randomly selected for RT-qPCR. The total RNA samples used for qPCR were the same for RNA-seq. The first strand of cDNA was synthesized by reverse transcription using the BioRT cDNA first strand synthesis kit (Bioer, Hangzhou, China) with oligo(dT) primer (Supplementary Table S1). Beta-actin, which showed similar FPKM values among treatments, was also included as the internal control for qPCR. The qPCR experiments were performed in 20 µl of reaction system using BioEasy master mix (Bioer, Hangzhou, China). The thermal cycles were performed and the signals of SYBR Green were detected using a Gene9600 Plus qPCR machine (Bioer, Hangzhou, China). Melting curves were prepared to reveal the specificity of primers. Expression levels of each gene were compared by calculating their relative fold change using the 2^−ΔΔCt^ method^30^.

## Results and Discussion

Preliminary experiments were conducted to test the tolerance of *C. auriculatum* to saline stress. Seedlings were treated with salinities ranging from 7.5‰ to 15‰. After treatment for one week, the leaves of *C. auriculatum* turned yellow, the whole plant was shorter and the bottom leaves withered in the 7.5‰ treatment, compared with the control. In treatments with 10‰, 12‰ and 15‰, the plants died (Fig. S1). These results indicated that *C. auriculatum* could tolerate 7.5‰ salinity. To explore the underlying molecular mechanisms, treatments with 3.75‰ and 7.5‰ were re-prepared for transcriptome sequencing.

### Illumina sequencing

The sequencing data have been deposited in National Center for Biotechnology Information (NCBI) with the bioproject number of PRJNA558052. RNA sequencing resulted in 65 M to 73 M of clean reads for each leaf sample. Q20 and Q30 values were all higher than 96.92% and 88.19%, respectively (Table S2). These results suggested that the quality of sequencing data could satisfy further transcriptome analyses.

### *De novo* assembly

Separate assembly of each sample was tried first. The results showed that the total number of unigenes ranged from 43,123 to 76,090, and the mean length of unigenes ranged from 1,257 bp to 1,521 bp. Alternatively, all sequencing data were assembled together, finally producing 196,199 unigenes with the mean length of 1,673 bp (Table S3). Among them, 15.89% unigenes were shorter than 300 bp and 17.07% were longer than 3,000 bp (Fig. S2). Benchmarking Universal Single-Copy Orthologs (BUSCO)^31^ analyses revealed that 99% BUSCOs were complete and 1% BUSCOs were missing (Fig. S3). Due to the longer mean length and higher ratio of BUSCOs, the unigene library assembled from nice samples together was used for the subsequent analyses.

### Prediction of coding sequences and functional annotation

Prediction of coding sequences detected 122,855 CDSs in total. Their total length and N50 length was 137.21 M bp and 1,431 bp, respectively (Table S4). Among them, 77.44% CDSs were longer than 500 bp, and 42.61% CDSs were longer than 1,000 bp (Fig. S4).

Blasting against the public databases, 73.35%, 64.41%, 57.08%, 59.70%, 60.62%, 57.71%, and 43.61% unigenes could be annotated to the NCBI non-redundant proteins (NR), nucleotide (NT), Swissprot, KEGG, Eukaryotic Orthologous Groups (KOG), Pfam, and GO databases, respectively (Table 1).

**Table 1:** BLAST analysis of non-redundant unigenes against public databases.

	Number of unigenes	Percentage (%)
Total unigenes	196,199	100
Annotated in NR	143,913	73.35
Annotated in NT	126,371	64.41
Annotated in Swissprot	111,994	57.08
Annotated in KEGG	117,134	59.70
Annotated in KOG	118,931	60.62
Annotated in Pfam	113,221	57.71
Annotated in GO	85,559	43.61
Intersection	48,166	24.55
Overall	159,285	81.19

### Differentially expressed genes and qPCR validation

**Figure 1:**
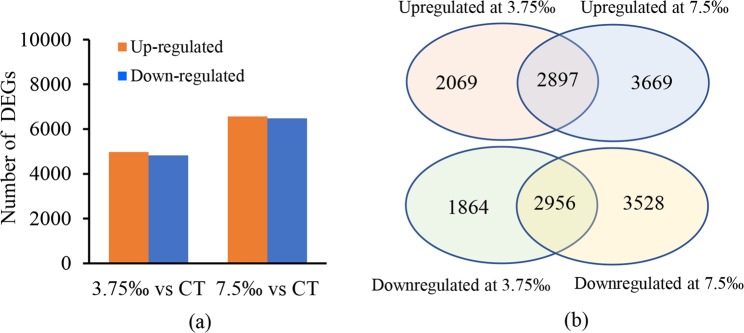
Differentially expressed genes (DEGs) in *C. auriculatum* leaves in response to saline stress. (**a**) Numbers of DEGs between treatment with 3.75‰ and 7.5‰ salinity. (**b**) Venn diagrams of up-regulated and down-regulated DEGs in treatments with 3.75‰ and 7.5‰. The numbers did not include genes with FPKM values lower than 2 in all samples. 3.75‰ vs CT, 3.75‰ salinity compared to the control; 7.5‰ vs CT, 7.5‰ salinity compared to the control.

Pairwise comparisons revealed that 16,849 and 19,404 unigenes were significantly up-regulated, 18,744 and 38,642 were down-regulated in treatment with 3.75‰ and 7.5‰, respectively, compared with the control. After removing unigenes with FPKM values lower than 2 in all samples, 4,975 and 6,566 unigenes were significantly up-regulated, 4,820 and 6,484 were significantly down-regulated in treatment with 3.75‰ and 7.5‰, respectively (Fig. 1a). Among them, 2,897 up-regulated DEGs and 2,956 down-regulated DEGs were commonly shared between treatments with 3.75‰ and 7.5‰ (Fig. 1b). The top 30 DEGs are listed in Table S5.

To validate the DEGs identified by RNA-Seq, nine randomly selected DEGs were determined by qPCR. The results showed similar changing trends between qPCR validation and FPKM calculation for all selected genes (Fig. S5), indicating that the transcriptome data were reliable.

### Enrichment of GO categories for DEGs

**Figure 2:**
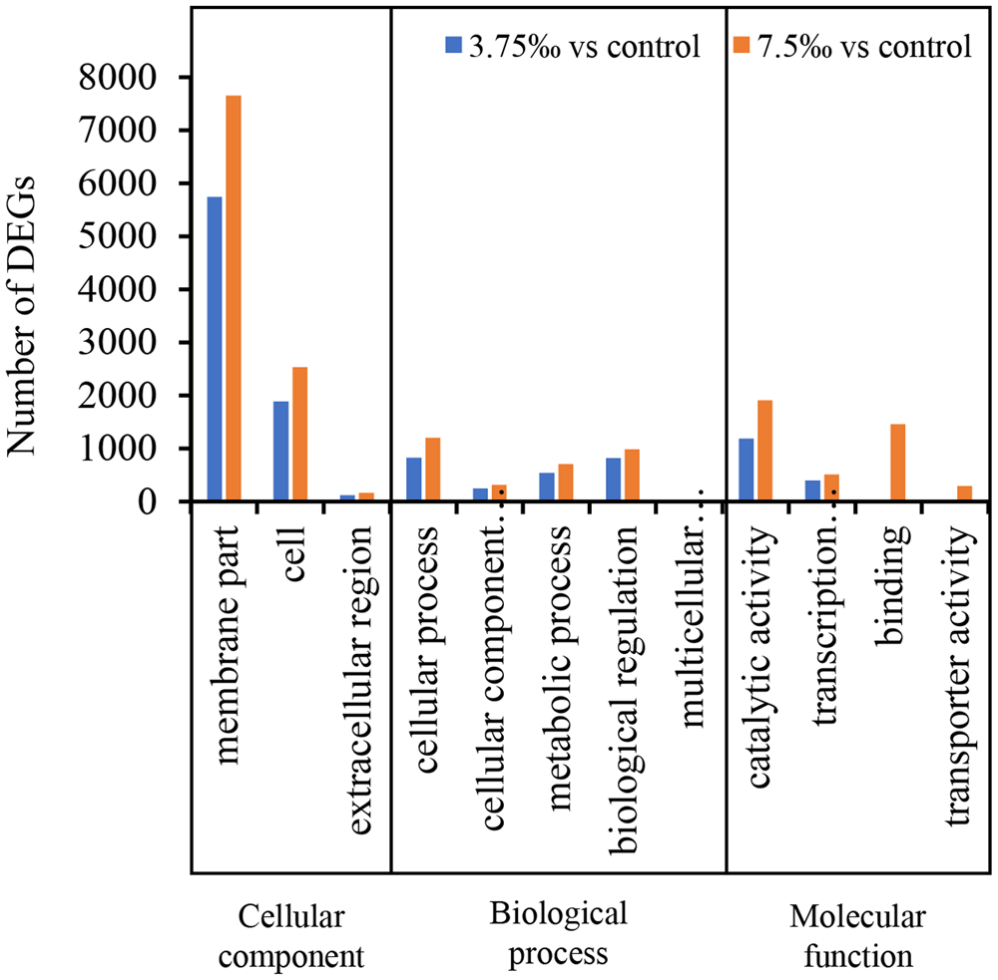
Top 12 GO categories of DEGs in *C. auriculatum* leaves in response to 3.75‰ and 7.5‰ saline stress.

In both treatments with 3.75‰ and 7.5‰, the top four GO categories were ‘membrane part’, ‘cell’, ‘catalytic activity’ and ‘cellular process’ (Fig. 2). In these categories, more DEGs were included in treatment with 7.5‰ than 3.75‰, suggesting that higher salinity caused more severe effects on *C. auriculatum* leaves. Besides, the category of ‘binding’ was only highly expressed in treatment with 3.75‰ but not in treatment with 7.5‰.

### Enrichment of KEGG pathways for DEGs

KEGG enrichment analysis of down-regulated genes in treatment with 3.75‰ and 7.5‰ revealed 19 and 28 pathways, respectively (Tables 2 and 3). These pathways might represent the inhibitory effects on the molecular processes in *C. auriculatum* leaves. Analysis of up-regulated genes, which represented mechanisms underlying resistance to saline stress, showed 19 and 23 significantly enriched pathways at 3.75‰ and 7.5‰, respectively, which shared 12 KEGG pathways (ko00500, ko00940, ko04075, ko00563, ko00941, ko00330, ko00511, ko00591, ko04016, ko00270, ko00460 and ko00904; Tables 2 and 3). These enriched pathways mainly involved in oxidative stress-related function, signal transduction, carbohydrate metabolism and lipid metabolism.

**Table 2:** Enrichment of KEGG pathway for differentially expressed genes between treatment with 3.75‰ and the control in *C. auriculatum*.

Pathway ID	Pathway Name	Enriched genes	P value	Q value
**Up-regulated genes in treatment with 3.75‰**				
ko00500	Starch and sucrose metabolism	411	9.76E-12	1.30E-09
ko04075	Plant hormone signal transduction	418	2.73E-11	1.82E-09
ko00563	Glycosylphosphatidylinositol (GPI)-anchor biosynthesis	82	8.44E-09	3.74E-07
ko00940	Phenylpropanoid biosynthesis	293	4.17E-06	9.24E-05
ko04016	MAPK signaling pathway - plant	334	4.66E-05	0.000886
ko00330	Arginine and proline metabolism	105	0.000198	0.00329
ko00511	Other glycan degradation	107	0.001072	0.012138
ko00591	Linoleic acid metabolism	81	0.001174	0.012138
ko00905	Brassinosteroid biosynthesis	37	0.001186	0.012138
ko00920	Sulfur metabolism	71	0.001029	0.012138
ko00941	Flavonoid biosynthesis	75	0.001069	0.012138
ko00904	Diterpenoid biosynthesis	55	0.001539	0.014622
ko03410	Base excision repair	83	0.00193	0.017116
ko02010	ABC transporters	156	0.002627	0.021835
ko00906	Carotenoid biosynthesis	84	0.003458	0.027052
ko00460	Cyanoamino acid metabolism	139	0.00424	0.031326
ko00270	Cysteine and methionine metabolism	166	0.006196	0.043373
ko00261	Monobactam biosynthesis	25	0.00766	0.048978
ko03430	Mismatch repair	75	0.007733	0.048978
**Down-regulated genes in treatment with 3.75‰**				
ko00460	Cyanoamino acid metabolism	181	3.78E-09	2.51E-07
ko00500	Starch and sucrose metabolism	402	2.13E-07	7.07E-06
ko00592	alpha-Linolenic acid metabolism	111	3.34E-06	8.87E-05
ko00906	Carotenoid biosynthesis	100	1.95E-05	0.00041
ko02010	ABC transporters	179	2.16E-05	0.00041
ko00770	Pantothenate and CoA biosynthesis	75	3.03E-05	0.000503
ko00790	Folate biosynthesis	67	5.80E-05	0.000858
ko00650	Butanoate metabolism	71	7.42E-05	0.000986
ko00591	Linoleic acid metabolism	89	0.000193	0.00233
ko00196	Photosynthesis - antenna proteins	23	0.000262	0.002871
ko04626	Plant-pathogen interaction	414	0.000281	0.002871
ko00564	Glycerophospholipid metabolism	159	0.001366	0.012981
ko00410	beta-Alanine metabolism	104	0.002037	0.018058
ko00051	Fructose and mannose metabolism	113	0.003898	0.027285
ko01210	2-Oxocarboxylic acid metabolism	115	0.003775	0.027285
ko04075	Plant hormone signal transduction	371	0.003377	0.027285
ko04712	Circadian rhythm - plant	135	0.003584	0.027285
ko00220	Arginine biosynthesis	86	0.005834	0.038797
ko00561	Glycerolipid metabolism	142	0.007597	0.048112

**Table 3:** Enrichment of KEGG pathway for differentially expressed genes between treatment with 7.5‰ and the control in *C. auriculatum*.

Pathway ID	Pathway Name	Enriched genes	P value	Q value
**Up-regulated genes in treatment with 7.5‰**				
ko00500	Starch and sucrose metabolism	467	1.25E-13	1.66E-11
ko00940	Phenylpropanoid biosynthesis	361	1.61E-11	1.07E-09
ko04075	Plant hormone signal transduction	453	1.25E-09	5.52E-08
ko00563	Glycosylphosphatidylinositol (GPI)-anchor biosynthesis	91	2.54E-09	7.49E-08
ko00941	Flavonoid biosynthesis	94	4.42E-06	8.41E-05
ko03030	DNA replication	101	6.71E-05	0.001116
ko00330	Arginine and proline metabolism	117	0.000148	0.002188
ko00511	Other glycan degradation	122	0.000337	0.004481
ko00591	Linoleic acid metabolism	92	0.000455	0.005505
ko04016	MAPK signaling pathway - plant	362	0.000562	0.006225
ko00945	Stilbenoid, diarylheptanoid and gingerol biosynthesis	49	0.000699	0.007147
ko00600	Sphingolipid metabolism	115	0.001326	0.012598
ko00270	Cysteine and methionine metabolism	190	0.002058	0.017484
ko00410	beta-Alanine metabolism	110	0.002103	0.017484
ko00460	Cyanoamino acid metabolism	157	0.002499	0.019553
ko00531	Glycosaminoglycan degradation	79	0.002989	0.022085
ko00592	alpha-Linolenic acid metabolism	101	0.003212	0.022486
ko00904	Diterpenoid biosynthesis	59	0.003443	0.022898
ko03430	Mismatch repair	85	0.004344	0.027509
ko00360	Phenylalanine metabolism	72	0.006695	0.037103
ko00520	Amino sugar and nucleotide sugar metabolism	234	0.006483	0.037103
ko00604	Glycosphingolipid biosynthesis - ganglio series	65	0.006317	0.037103
ko00260	Glycine, serine and threonine metabolism	117	0.009135	0.048599
**Down-regulated genes in treatment with 7.5‰**				
ko00052	Galactose metabolism	251	1.05E-06	4.74E-05
ko04712	Circadian rhythm - plant	218	1.57E-06	5.29E-05
ko00195	Photosynthesis	88	2.94E-06	7.94E-05
ko00906	Carotenoid biosynthesis	139	1.90E-05	0.000427
ko00630	Glyoxylate and dicarboxylate metabolism	286	6.28E-05	0.001212
ko02010	ABC transporters	249	9.48E-05	0.0016
ko00909	Sesquiterpenoid and triterpenoid biosynthesis	65	0.000108	0.001622
ko00790	Folate biosynthesis	90	0.000162	0.002186
ko00945	Stilbenoid, diarylheptanoid and gingerol biosynthesis	66	0.000226	0.002715
ko00966	Glucosinolate biosynthesis	18	0.000241	0.002715
ko00196	Photosynthesis - antenna proteins	30	0.000296	0.002854
ko00650	Butanoate metabolism	95	0.000295	0.002854
ko00460	Cyanoamino acid metabolism	220	0.00037	0.003121
ko00900	Terpenoid backbone biosynthesis	134	0.000348	0.003121
ko00500	Starch and sucrose metabolism	542	0.000405	0.003216
ko00591	Linoleic acid metabolism	122	0.000504	0.003783
ko00604	Glycosphingolipid biosynthesis - ganglio series	93	0.000542	0.00385
ko00531	Glycosaminoglycan degradation	109	0.000842	0.005411
ko00940	Phenylpropanoid biosynthesis	418	0.000808	0.005411
ko00061	Fatty acid biosynthesis	103	0.001988	0.012199
ko00770	Pantothenate and CoA biosynthesis	93	0.002773	0.016275
ko04146	Peroxisome	292	0.00293	0.016479
ko03450	Non-homologous end-joining	34	0.003761	0.020307
ko00603	Glycosphingolipid biosynthesis - globo and isoglobo series	37	0.004752	0.024675
ko00071	Fatty acid degradation	139	0.006563	0.032814
ko00230	Purine metabolism	466	0.007961	0.038381
ko00240	Pyrimidine metabolism	380	0.009931	0.046232
ko00780	Biotin metabolism	48	0.010923	0.049154

### Ion transportation

Under saline conditions, the first response of plants is ion toxicity, and generally Na^+^ displays more severe influence than Cl^−^, since Na^+^ would reach toxic concentrations earlier than Cl^−^. Thus, most studies focused on Na^+^ uptake, transportation and exclusion in plants^28^. In the present study, compared with the control, Na^+^/H^+^ exchanger was significantly up-regulated in treatment with 3.75‰ (P < 0.05), but K^+^ efflux antiporter, cation/H^+^ antiporter and sodium/proton antiporter were significantly down-regulated at 7.5‰ salinity (P < 0.05). V-type proton ATPase (V-ATPase) was significantly up-regulated, while cyclic nucleotide-gated ion channel (CNGC) were significantly down-regulated at 3.75‰ salinity (P < 0.05) (Table 4). The up-regulation of Na^+^/H^+^ exchanger might alleviate cytosolic salt accumulation in the vacuole and other cellular compartments^32,33^. Similar results were observed in *Arabidopsis*^34^, which assisted Na^+^ extrusion from plant cells. Activation of V-ATPase in plants might reduce Na^+^ accumulation by interacting with H^+^ pumps to resist saline stress^35^. CNGCs are non-selective ion channels involved in mediation of Na^+^ transport. The expression level of CNGC significantly decreased at 3.75‰ salinity compared with the control, which was in agreement with the findings reported in *Arabidopsis* that CNGC10 expression was inhibited when exposed to high salinity or low salinity for long time^36^. K^+^ efflux antiporter was significantly down-regulated at 7.5‰ salinity, similar to the transcriptome analysis of grapevine (*Vitis vinifera* L.) under saline conditions^37^, implying the replacement of K^+^ by Na^+^.

**Table 4:** FPKM values of selected genes in saline treatment and the control in *C. auriculatum*.

Gene name	Control	3.75‰	7.5‰
**Antioxidant enzymes**			
Superoxide dismutase [Cu-Zn]	235.51 ± 24.41	264.66 ± 10.57	289.7 ± 28.82
Superoxide dismutase [Mn]	54.59 ± 10.99	72.45 ± 12.22	117.67 ± 31.78*
Superoxide dismutase [Fe]	28.52 ± 2.81	54.61 ± 12.01*	45.86 ± 18.52
Copper chaperone for superoxide dismutase	7.34 ± 1.45	7.54 ± 1.28	6.95 ± 2.10
**Flavonoid**			
Chalcone synthase (CHS)	68.77 ± 9.01	89.72 ± 21.40	93.91 ± 64.69
Chalcone isomerase (CHI)	19.59 ± 3.36	27.24 ± 6.99	32.4 ± 11.73*
Flavonol synthase (FLS)	13.31 ± 2.75	14.32 ± 1.78	15.66 ± 6.44
Trans-cinnamate 4-monooxygenase (C4H)	13.68 ± 2.29	25.12 ± 3.85*	34.80 ± 17.19*
Bifunctional dihydroflavonol 4-reductase (DFR)	42.88 ± 1.63	44.43 ± 12.11	38.85 ± 2.82
**Phenylpropanoid**			
Phenylalanine ammonia-lyase (PAL)	16.98 ± 1.38	24.99 ± 6.69*	36.10 ± 17.76*
4-coumarate–CoA ligase (4CL)	26.64 ± 1.96	25.73 ± 6.86	39.11 ± 17.74
Carotenoid			
Phytoene synthase (PSY)	38.58 ± 6.00	27.06 ± 5.75	25.08 ± 7.01
Phytoene desaturase (PDS)	2.70 ± 0.29	2.79 ± 1.05	0.75 ± 0.27*
Zeta-carotene desaturase (Zds)	41.30 ± 3.65	45.52 ± 13.66	40.05 ± 13.97
Nine-cis-epoxycarotenoid dioxygenase (NCED)	10.02 ± 2.77	3.89 ± 0.86*	9.11 ± 2.93
Beta-carotene 3-hydroxylase (CrtR-b)	3.31 ± 1.02	3.32 ± 0.92	3.19 ± 1.15
**Ion transprotation**			
Sodium/hydrogen exchanger	83.49 ± 2.97	93.32 ± 5.81*	95.84 ± 16.92
K^+^ efflux antiporter	147.81 ± 8.44	124.32 ± 22.92	110.41 ± 19.92*
Cation/H^+^ antiporter	26.04 ± 3.85	22.68 ± 1.18	19.55 ± 4.05*
Sodium/proton antiporter	4.61 ± 1.34	4.52 ± 0.57	2.83 ± 0.51*
V-type proton ATPase (V-ATPase)	1022.52 ± 68.32	1200.48 ± 109.60*	1315.03 ± 167.55
Cyclic nucleotide-gated ion channel (CNGCs)	182.29 ± 14.62	124.27 ± 7.17*	162.24 ± 16.93
Potassium channel	143.87 ± 28.09	127.9 ± 2.31	150.15 ± 10.90
Two-pore potassium channel	24.78 ± 2.70	22.58 ± 0.62	26.38 ± 1.61
Chloride channel protein (CLC)	167.38 ± 29.56	152.79 ± 8.84	138.88 ± 8.4
Choline monooxygenase (CMO)	24.10 ± 5.08	21.21 ± 7.30	21.44 ± 7.01
**Lipid metabolisms**			
Lipoxygenase	40.09 ± 6.61	19.49 ± 5.35*	26.36 ± 11.97
Hydroperoxide dehydratase	82.68 ± 49.35	19.90 ± 0.62*	40.49 ± 9.74
Allene oxide cyclase	40.38 ± 5.28	41.88 ± 5.00	62.92 ± 15.02
12-oxophytodienoic acid reductase	79.17 ± 11.09	93.19 ± 15.21	87.5 ± 4.66

Data represent mean ± standard deviation of FPKM values (n = 3). *Significantly different from the control (P < 0.05, Student’s t-test).

### Saline treatments induced antioxidant mechanisms in *C. auriculatum*

Oxidative stress is a general negative effect on plants caused by saline stress. To resist saline stress, antioxidant mechanisms were activated^38^. Among them, SOD is an important antioxidant enzyme^39^. SODs have three isozymes including copper-zinc SOD (Cu/Zn-SOD), manganese SOD (Mn-SOD) and iron SOD (Fe-SOD). In the present study, three isozymes were all detected. In comparison to the control, Mn-SOD was significantly up-regulated for 2.15 times in 7.5‰ treatment, while Fe-SOD increased significantly for 1.91 times at 3.75‰ salinity (P < 0.05) (Table 4). These changes might enhance tolerance of *C. auriculatum* to saline stress, since it has been reported that overexpression of Mn-SOD enhanced salt-tolerance in *Arabidopsis*^40^. In pea, the activity of Mn-SOD in salt-tolerant plants is higher than that in salt-sensitive plants under NaCl treatment. Further analysis found that NaCl-tolerant plants had protective mechanisms against salt-induced O_2_^−^ production by increasing the mitochondrial Mn-SOD activity^41^. According to these results, *C. auriculatum* might be salt-tolerant, but the specific functions of SODs need further study.

Flavonoid biosynthesis pathway plays a pivotal role and protects the plant cells from oxidative damage by scavenging free radicals^42,43^. KEGG enrichment analyses of up-regulated DEGs significantly enriched flavonoid biosynthesis pathway. Within this pathway, trans-cinnamate 4-monooxygenase (C4H) and chalcone isomerase (CHI) are key enzymes, directly related to the synthesis of flavonoids^44^. In the present study, compared with the control, CHI and C4H were significantly up-regulated in treatments with 3.75‰ and/or 7.5‰ (Table 4). These results implied that more flavonoids were synthesized, which might contribute to the total antioxidant capacity in response to saline stress in *C. auriculatum*. Similarly, Walia *et al*.^45^ reported that a large number of genes were up-regulated in the flavonoid biosynthesis pathway under salinity stress, which played an important protective role against saline stress.

Phenylpropanoids are involved in antioxidant activity in cell walls and lignin biosynthesis in secondary metabolites, and play an important role in plant response to abiotic stress, which connects to flavonoid biosynthesis^46^. Phenylalanine ammonia-lyase (PAL) is the first enzyme that converts phenylalanine into cinnamic acid and is transcriptionally regulated in response to many environmental cues^47^. PAL was a marker during molecular breeding to improve resistance of plants to injury and saline stress^48^. In the present study, PAL was significantly up-regulated for 1.47 and 2.13 times at 3.75‰ and 7.5‰ salinity (P < 0.05), respectively, compared with the control (Table 4). Similar findings were observed in salinity-stressed *Olea europaea*^49^.

Carotenoids reveal antioxidant activity in plants. In the carotenoid biosynthesis pathways, phytoene synthase (PSY) is a rate-limiting enzyme, related to abiotic stress and mainly involved in plant stress response^50^. In the present study, compared with the control, the expression level of phytoene desaturase (PDS) was significantly lower in treatment with 7.5‰ and that of nine-cis-epoxycarotenoid dioxygenase (NCED) was significantly lower in treatment with 3.75‰. Besides, the expression level of phytoene synthase (PSY) was lower in saline treatments than that in the control, although no statistical significance was detected (P > 0.05; Table 4). Similarly, *PSY1* gene was reduced in response to enhanced NaCl level^51^, and *NCED* gene was clearly repressed under drought and saline stress in cotton^52^. The decreased expression of key enzymes in carotenoid biosynthesis pathway revealed that carotenoid biosynthesis might not be the antioxidant mechanism in salinity-stressed *C. auriculatum*.

### Saline treatments regulated lipid metabolism in *C. auriculatum*

Six lipid metabolism-related pathways were significantly enriched when up-regulated or down-regulated genes were subjected to KEGG enrichment analyses, including alpha-linolenic acid (LnA) metabolism, linoleic acid (LA) metabolism, glycerophospholipid metabolism, fatty acid biosynthesis, fatty acid degradation, and sphingolipid metabolism. In response to adverse factors, alpha-linolenic acid and linoleic acid were released and became the initial step of unsaturated fatty acid metabolism^53,54^. LnA was catalyzed by different functional lipoxygenase (LOX) to produce hydroperxy linolenic acid (HPOT), and then metabolized into a series of compounds with certain biological activities, such as jasmonic acid (JA), 3-hexenol and traumatic acid, which related to plant defense responses to abiotic stresses^54–56^. Previous studies have shown that JA biosynthesis genes were up-regulated in *Arabidopsis* and sunflower roots in saline treatments^57,58^. In the present study, compared with the control, LOX and hydroperoxide dehydratase were significantly down-regulated at 3.75‰ (P < 0.05) (Table 4), revealing that unsaturated fatty acid metabolism might be inhibited by saline stress in *C. auriculatum*.

### Changes of plant hormone signaling transduction pathways under saline stress

Plants respond to abiotic stress through a complex coordination of various phytohormone signaling pathways, mainly including auxin (AUX), cytokinine (CTK), gibberellin (GA), abscisic acid (ABA), ethylene (ETH), brassinosteroid (BR), jasmonic acid (JA) and salicylic acid (SA)^59^. In the present study, the functional analysis of DEGs by KEGG enrichment analysis showed that a large number of genes were involved in plant hormone signaling transduction and hormone responses under saline stress, highlighting the key roles of plant hormones in regulating *C. auriculatum* response to saline stress. In the JA signal pathway, the expression level of jasmonate ZIM domain-containing protein (JAZ) was significantly down-regulated at 3.75‰ salinity (Table S6), indicating that JA signaling transduction was inhibited by saline stress. This result was consistent with the inhibition of unsaturated fatty acid metabolism.

As previously reported, the BR signaling pathway was activated under saline stress in *Arabidopsis*^60^, and the exogenous application of BR effectively alleviated the adverse effects of abiotic stress or increased plant resistance to stresses^61,62^. In the present study, compared with the control, brassinosteroid insensitive 1 (BRI1), BR-signaling kinase (BSK), protein brassinosteroid insensitive 2 (BIN2), brassinosteroid resistant 1/2 (BZR1/2), xyloglucan: xyloglucosyl transferase (TCH4) and cyclin-D3 (CYCD3) were significantly up-regulated in treatments with 3.75‰ and/or 7.5‰ (Table S6), suggesting that activation of BR signaling pathway might participate in resistance to saline stress.

**Figure 3:**
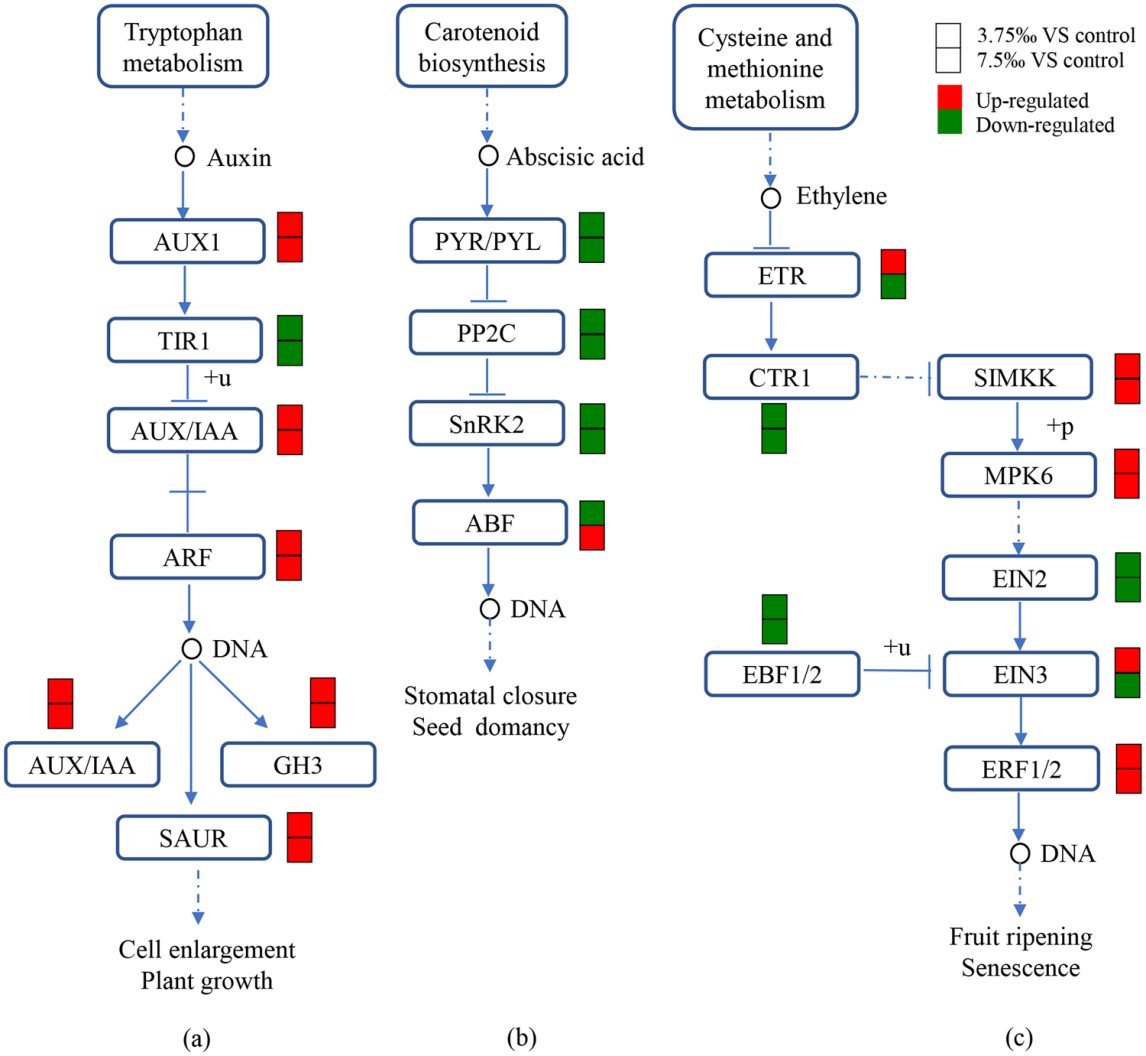
DEGs mapped to the plant hormone signaling transduction pathways in leaves. (**a**) Auxin signaling pathway, (**b**) ABA signaling pathway and (**c**) ethylene signaling pathway. Upper and lower color box indicate difference between 3.75‰ and the control, between 7.5‰ and the control, respectively. Red: up-regulated compared with the control. Green: down-regulated compared with the control.

Auxin can induce expression of auxin-response factor gene (ARF), auxin early response gene (Aux/IAA), gretchen hagen3 (GH3) and small auxin-up RNAs (SAUR) rapidly and instantaneously^63^. In the present study, compared with the control, GH3 and SAUR were significantly up-regulated in response to saline treatments. However, the expression levels of protein transport inhibitor response (TIR1) was significantly down-regulated under saline stress (P < 0.05; Fig. 3). Since GH3 and SAUR were downstream genes of auxin signaling pathway, the present results suggested that auxin signaling pathway was activated in *C. auriculatum* in response to saline stress.

The abscisic (ABA) signaling pathway is well known to be associated with responses to saline stress in plants and the increase of ABA content could help plants adapt and survive under saline condition by reducing the accumulation of Na^+^ and improving osmotic adjustment^15^. Besides, carotenoid biosynthesis pathway provides precursors for plant hormone abscisic acid (ABA) synthesis^64^. In the present study, ABA receptors (PYR/PYL) were significantly down-regulated in treatment with 7.5‰ (Fig. 3), which might be resulted from the inhibition of carotenoid biosynthesis. Similar results were also detected in *Corchorus* spp. and *Vitis vinifera* L^37,65^. Besides, downstream of PYR/PYL, serine/threonine-protein kinase 2 (SNRK2) was also significantly down-regulated for 2.03 and 1.97 times in treatment with 3.75‰ and 7.5‰, respectively (P < 0.05; Fig. 3). Overall, these results suggested that ABA signaling pathway was inhibited by saline treatments in *C. auriculatum*.

Ethylene is a stress hormone regulating myriad stress responses^66^. Ethylene receptors (ETR) serve as negative regulators of the ethylene signaling transduction pathway, while ethylene-insensitive protein 2 (EIN2) and ethylene-insensitive protein 3 (EIN3) are positive regulators. Thus, a decrease in receptor levels is predicted to sensitize the plant such that it responds to lower levels of ethylene than usual^67^. In the present study, compared with the control, mitogen-activated protein kinase kinase (SIMKK) and ethylene-responsive transcription factor 1/2 (ERF1/2) were significantly up-regulated in treatment with 7.5‰, while serine/threonine-protein kinase CTR1 (CTR1) and EIN3-binding F-box protein 1/2 (EBF1/2) were significantly down-regulated in treatment with 3.75‰ and 7.5‰, respectively (P < 0.05; Fig. 3, Table S6). No significant differences in expression levels of ETR, EIN2 and EIN3 were detected between saline treatments and the control. Considering ERF1/2 is a downstream gene of CTR1 and EBF1/2 (Fig. 3), the present results implied that ethylene signaling pathway might be activated in *C. auriculatum* under saline conditions.

In summary, the changes of gene expression profile in *C. auriculatum* leaves under saline stress were explored using the transcriptome sequencing to investigate regulatory mechanisms in response to saline stress. Compared with the control, *C. auriculatum* showed suppressed carotenoid biosynthesis, lipid metabolisms, abscisic acid and jasmonic acid signaling transduction, which might damage plant cells. Expression levels of Na^+^/H^+^ exchanger and V-type proton ATPase, the flavonoid and phenylpropanoids biosynthesis pathways, the auxin and ethylene signaling pathways were upregulated to improve the antioxidant capacity of *C. auriculatum* in response to saline treatments. These changes might facilitate resistance of *C. auriculatum* to saline stress. Flavonoid and phenylpropanoids appear to play roles in resistance to salt tolerance in *C. auriculatum*, possibly due to its pharmacodynamics.

## Supplementary information

supplementary information.

## Data Availability

The original sequencing files have been deposited in the National Center for Biotechnology Information (NCBI) with the bioproject number of PRJNA558052.
