# Supplementary material for: Transcriptome analyses revealed molecular responses of Cynanchum auriculatum leaves to saline stress
Source: Sci Rep. 2020 Jan 16;10:449. doi: 10.1038/s41598-019-57219-8 (PMC6965089; doi:10.1038/s41598-019-57219-8)
Supplement: Supplementary file 1 — supplementary information. [file 41598_2019_57219_MOESM1_ESM.pdf]

Supplementary information:

Transcriptome analyses revealed molecular responses *Cynanchum auriculatum* leaves to saline stress

Ming Zhang[1#], Li-Zhou Hong[1#], Min-Feng Gu[1#], Cheng-Dong Wu[1*], Gen Zhang[2*]

[1] Xinyang Agricultural Experiment Station of Yancheng City, Jiangsu Province, 224045, P. R. China

[2] Shenzhen GenProMetab Biotechnology Company Limited., Shenzhen, Guangdong Province, 51800, P. R. China

# These three authors contribute equally to this work.

* correspondence to:

Mr. Cheng-Dong Wu, syzwcd@126.com

Dr. Gen Zhang, zhanggen1988@163.com

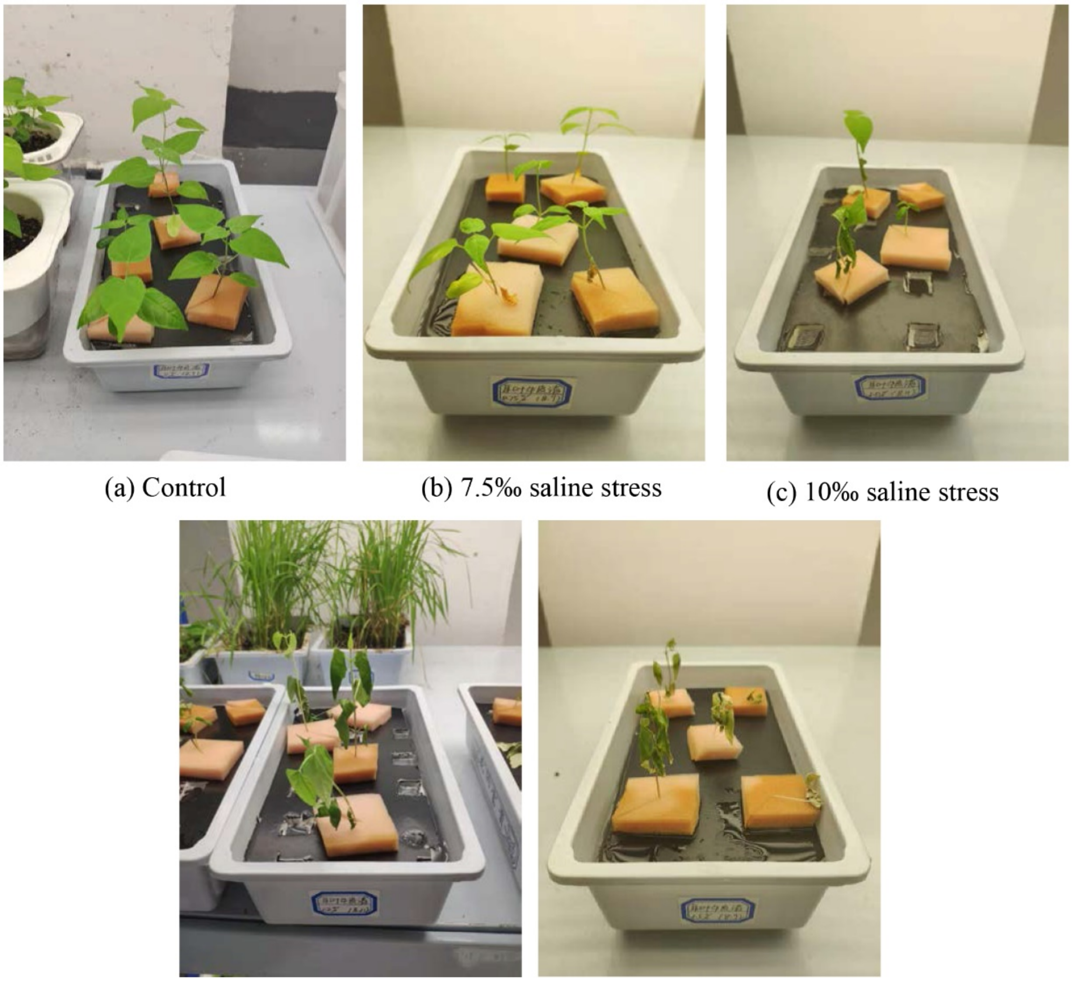

(a) Control (b) 7.5‰ saline stress (c) 10‰ saline stress

(d) 12‰ saline stress (e) 15‰ saline stress

Figure S1. Phenotypic changes of leaves under saline stress after one week

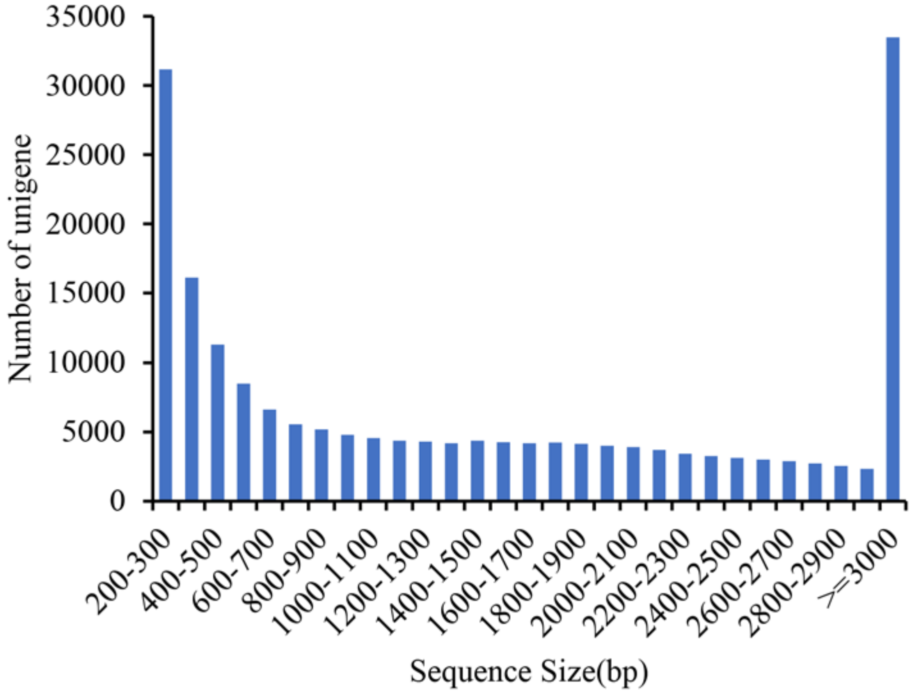

Figure S2. Length distribution of unigenes.

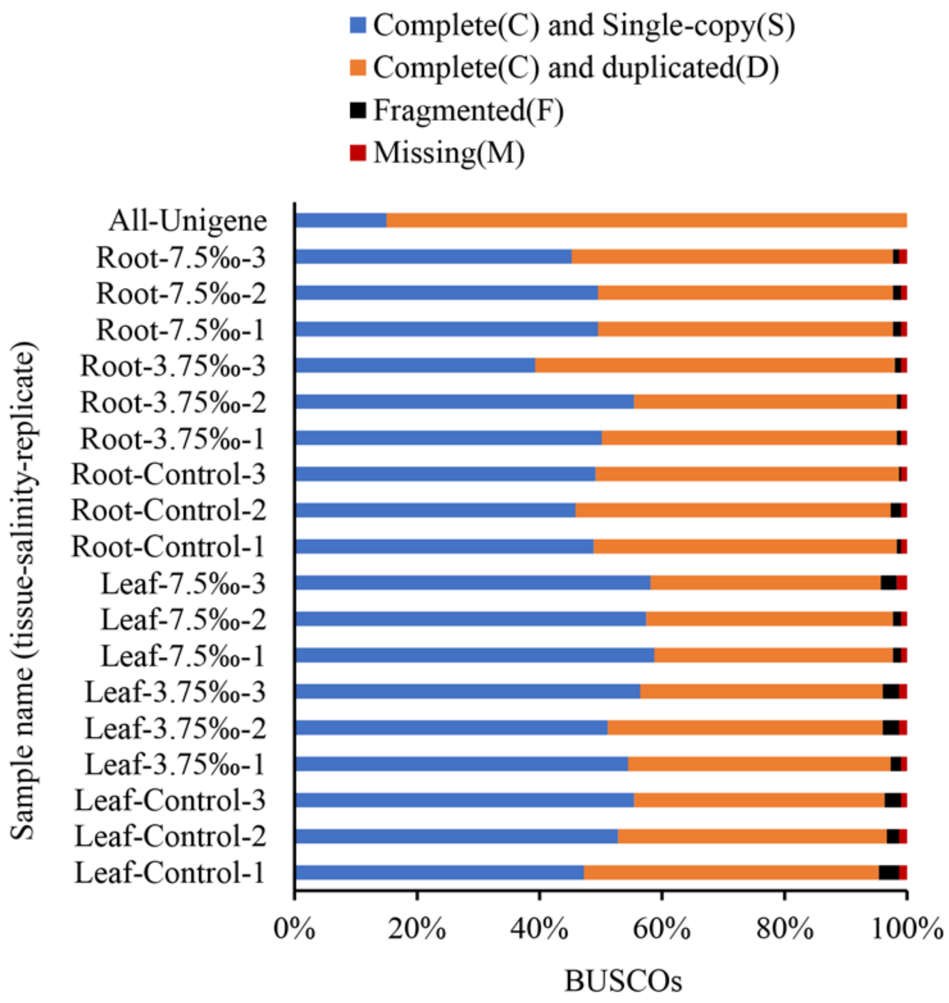

Figure S3. Results of BUSCO analyses.

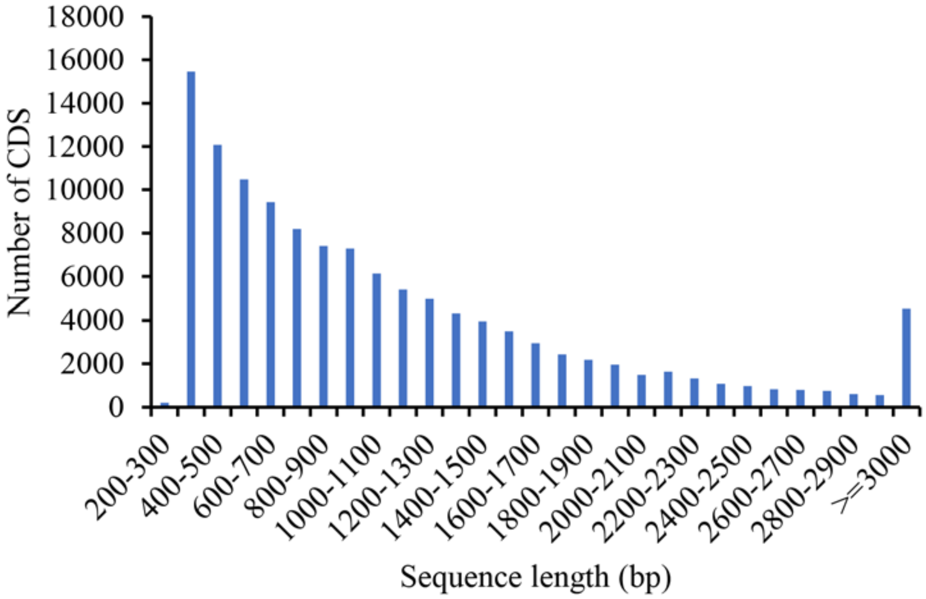

Figure S4. Length distribution of CDS.

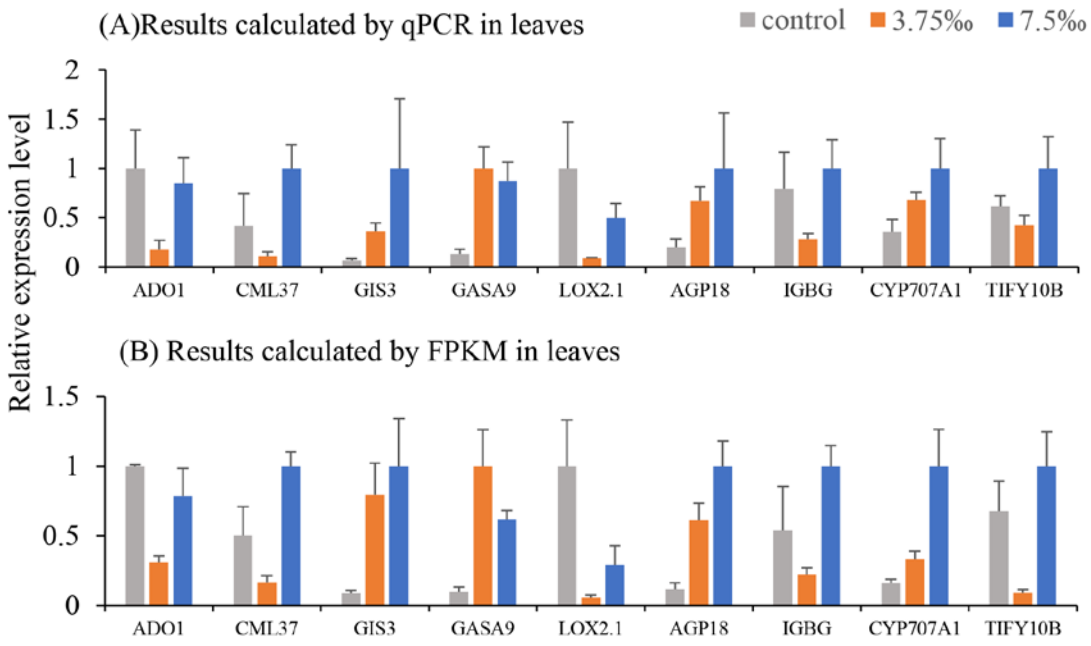

Figure S5. Real-time qPCR validation of Illumina sequencing results. The highest value among treatments and the control was normalized as one for each gene. Date represent mean ± SD. ADO1: Adagio protein 1; CML37: Calcium-binding protein CML37; GIS3: Zinc finger protein GIS3; GASA9: Gibberellin-regulated protein 9; LOX2.1: Linoleate 13S-lipoxygenase 2-1 chloroplastic; AGP18: Lysine-rich arabinogalactan protein 18; IGBG: Isoflavonoid 7-O-beta-apiosyl-glucoside beta-glycosidase, CYP707A1: Abscisic acid 8'-hydroxylase 1; TIFY10B: Protein TIFY 10b.

Table S1. Primers used in the present study.

Gene description	Direction	Primer sequence (5' to 3')
Adagio protein 1	Forward	TGGCAGCAATACTGGTGGAG
	Reverse	GCAGGATATGCACCTACGCT
Calcium-binding protein CML37	Forward	TCCTCCAAACAAAGCGACCC
	Reverse	AAAGTATCGCCGAGCGAGTT
Zinc finger protein GIS3	Forward	CCGCGTACTCTCTCCGTTAC
	Reverse	TGACGACTCCTCTTCCGAGT
Gibberellin-regulated protein 9	Forward	AAGGAGATGCAGCAAGGCAT
	Reverse	CATAGCAGGGACAAGCCTCC
Linoleate 13S-lipoxygenase 2-1 chloroplastic	Forward	CGACCGTGCGAAGGATGATA
	Reverse	ATCGTGACTGCTTTTCCGGT
Lysine-rich arabinogalactan protein 18	Forward	CGGCTCCAGCTCCATCTAAG
	Reverse	GACCAAGGAGGTCAGGTGTG
Isoflavonoid 7-O-beta-apiosyl-glucoside beta-glycosidase	Forward	TAAGCAGCAGAGGCAAGACC
	Reverse	CACAGGTCCAACTCCTCGTC
Abscisic acid 8'-hydroxylase 1	Forward	GGGATGTCCATGCGTGATGA
	Reverse	GGGTGAATGCTCGGAGAACA
Protein TIFY 10b	Forward	ACCTCTTTACCCCCGGATGA
	Reverse	TGAAACTGAGGCCCGATTCC
Stem-specific protein TSJT1	Forward	ACACCCAGCAGGAAATGGAG
	Reverse	GGGCATAGCTGCTGATGGAT
40S ribosomal protein S25	Forward	GGAGGTTCATCTGGTGGTGG
	Reverse	ACGAGCAAGTGAACCACGAA
60S ribosomal protein L38	Forward	AACCGATTTGGCGTCTTTGC
	Reverse	TCAACTCGAACGCATCACCA
40S ribosomal protein S9-1	Forward	ATTCGGACGAGACGACGAAG
	Reverse	AAAGATTCGCAAAGCTGCCC
40S ribosomal protein S2-1	Forward	ACACCGAGACCAATGTGTCC
	Reverse	GAAACAAACCCGTGCTGGTC
Cytochrome b	Forward	TCGAGAGGGGCAAGTTTGTT
	Reverse	GCTCAAAATGATATCTGCCCCC
V-type proton ATPase subunit C	Forward	ACCCCATCAACGGAGAGAGA
	Reverse	AGATACGGCGCCAGATTGAG
Heat shock protein 90-1	Forward	CACGCTTTGGGACGAACAAG
	Reverse	CCTTGTCCAACGACTGGGAA
Heat shock cognate protein 80	Forward	CTGAGTCGCCACTTTCGAGT
	Reverse	GGCAAGTGGTTTCGCTCTTG
β-actin	Forward	GGACCGGACTCGTCGTATTC
	Reverse	CACAGCCCTTGCTCCAAGTA

Table S2. Summary of clean data of transcriptome sequencing

Sample		Total Reads	Total Bases	Q20 (%)	Q30 (%)	Clean Reads Ratio (%)
control	1	73.78	7.38	97.06	88.63	95.57
	2	68.94	6.89	97.09	88.73	95.46
	3	68.66	6.87	97.09	88.78	92.37
3.75‰	1	68.53	6.85	97.07	88.75	94.89
	2	65.22	6.52	97	88.47	94.97
	3	66.4	6.64	97.02	88.49	95.22
7.5‰	1	70.92	7.09	96.92	88.19	94.92
	2	66.16	6.62	97.03	88.59	94.87
	3	69.99	7	98.09	90.88	96.91

Table S3. Statistics of unigenes assembled based on each sample and all samples mixed together

Sample		TN	TL (M)	ML	N50	N70	N90	GC (%)
Control	1	76090	99.71	1310	2213	1509	551	38.64
	2	59635	79.45	1332	2099	1489	655	39.32
	3	60113	82.89	1378	2190	1539	682	39.35
3.75‰	1	57685	78.85	1366	2151	1512	680	39.45
	2	62607	78.75	1257	2051	1412	553	39.17
	3	52784	72.00	1363	2118	1509	689	39.49
7.5‰	1	43123	65.60	1521	2188	1588	830	39.84
	2	47593	70.29	1476	2193	1570	790	39.72
	3	47323	71.42	1509	2174	1577	825	39.72

TN: total number; TL: total length; ML: mean length

Table S4. Quality Indicators of CDS

TN	TL (M)	N50	N90	Max Length	Min Length	GC (%)
122855	137.21	1431	552	15345	297	42.78

TN: total number; TL: total length

Table S5. The regulation of top 30 DEGs in treatment with 3.75‰ and 7.5‰ salinity compared with control.

Gene ID	Anotation	Log_2FC	Q value	Regulated
3.75‰ VS CT				
Unigene17934_All	Protein CURVATURE THYLAKOID 1A, chloroplastic	-15.23	0	down
CL4485.Contig7_All	Acetylornithine deacetylase	-14.57	0	down
Unigene50242_All	Protein NRT1/ PTR FAMILY 2.11	13.75	0	up
CL753.Contig5_All	Heat shock cognate 70 kDa protein 2	-13.32	0	down
CL14598.Contig29_All	Protein TIC 100	-13.29	0	down
CL730.Contig32_All	NA	-13.13	0	down
Unigene46185_All	UBP1-associated protein 2A	-13.11	0	down
CL8117.Contig5_All	26S proteasome regulatory subunit 7 homolog A	13.00	0	up
CL1465.Contig14_All	Probable transcription factor At4g00390	12.96	0	up
CL14718.Contig17_All	NA	12.90	0	up
CL1236.Contig24_All	Serine/arginine-rich splicing factor SR45a	-12.89	0	down
CL768.Contig17_All	Light-harvesting complex-like protein OHP1, chloroplastic	-12.88	0	down
CL353.Contig3_All	Thioredoxin reductase NTRC	12.84	0	up
CL439.Contig19_All	Uncharacterized protein At1g04910	12.82	0	up
CL2443.Contig2_All	Superoxide dismutase [Fe], chloroplastic (Fragment)	12.80	0	up
Unigene27288_All	GDP-L-galactose phosphorylase 2	-12.7	0	down
CL4360.Contig8_All	Beta-Amyrin Synthase 1	-12.67	0	down
CL1004.Contig53_All	Protein trichome birefringence-like 37	-12.61	0	down
CL2334.Contig5_All	Protein NCA1	12.61	0	up
CL14771.Contig8_All	Ion channel CASTOR	12.59	0	up
Unigene26048_All	Carbonic anhydrase, chloroplastic	12.53	0	up
CL730.Contig33_All	Protein Ycf2	-12.5	0	down
CL11956.Contig38_All	Dihydropyrimidinase	-12.48	0	down
CL1236.Contig43_All	Alpha-glucan phosphorylase, H isozyme	-12.45	0	down
Unigene44690_All	S-type anion channel SLAH3	12.44	1.39E-293	up
Unigene29898_All	2-carboxy-D-arabinitol-1-phosphatase	-12.43	0	down
Unigene33904_All	Ribosome-recycling factor,	-12.33	3.23E-	down

	chloroplastic (Fragment)		293	
CL6763.Contig11_All	bZIP transcription factor 46	-12.33	3.78E-292	down
CL1921.Contig4_All	Glycine-rich cell wall structural protein 1.0	12.30	1.61E-273	up
7.5‰ VS CT				
Unigene17934_All	Protein CURVATURE THYLAKOID 1A, chloroplastic	-15.29	0	down
Unigene50242_All	Protein NRT1/ PTR FAMILY 2.11	14.65	0	up
CL1967.Contig14_All	Cathepsin B-like protease 3	14.13	0	up
Unigene34624_All	Phosphomethylpyrimidine synthase, chloroplastic	-14.13	0	down
Unigene44794_All	Zinc finger A20 and AN1 domain-containing stress-associated protein 8	13.74	0	up
CL1921.Contig4_All	Glycine-rich cell wall structural protein 1.0	13.33	0	up
CL730.Contig32_All	NA	-13.19	0	down
Unigene87467_All	4-hydroxy-7-methoxy-3-oxo-3,4-dihydro-2H-1,4-benzoxazin-2-yl glucoside beta-D-glucosidase 1c, chloroplastic	13.18	0	up
CL8749.Contig1_All	Protein FREE1	13.14	0	up
CL3451.Contig24_All	Glutamyl-tRNA reductase 1, chloroplastic	13.08	0	up
CL4462.Contig6_All	Protein PLASTID TRANSCRIPTIONALLY ACTIVE 12	12.86	0	up
Unigene43459_All	H/ACA ribonucleoprotein complex subunit 4	12.74	0	up
CL1921.Contig9_All	Transcription initiation factor TFIID subunit 15b	-12.74	0	down
CL4187.Contig24_All	Xyloglucan endotransglucosylase/hydrolase protein 15	12.72	0	up
CL1921.Contig29_All	Transcription initiation factor TFIID subunit 15b	-12.69	0	down
Unigene23157_All	Low-temperature-induced 65 kDa protein	12.64	0	up
CL730.Contig33_All	Protein Ycf2	-12.56	0	down
Unigene45399_All	NA	-12.56	0	down
Unigene26326_All	Serine decarboxylase	12.56	0	up
CL1236.Contig43_All	Alpha-glucan phosphorylase, H isozyme	-12.51	0	down

CL14904.Contig2_All	PTI1-like tyrosine-protein kinase 3	12.50	0	up
CL439.Contig19_All	Uncharacterized protein At1g04910	12.48	0	up
Unigene26330_All	Serine decarboxylase	-12.44	3E-303	down
Unigene48166_All	ATP-citrate synthase alpha chain protein 2	12.44	3.8E-300	up
CL353.Contig3_All	Thioredoxin reductase NTRC	12.39	8.3E-294	up
Unigene25889_All	Mannose-1-phosphate guanylyltransferase 1	12.36	0	up
CL6549.Contig2_All	La-related protein 6A	-12.34	5.8E-289	down
CL8031.Contig6_All	Malate dehydrogenase [NADP], chloroplastic	-12.32	1.1E-285	down
Unigene26659_All	Probable WRKY transcription factor 19	12.31	1.4E-281	up

Table S6. FPKM values of genes involved in phytohormone signal transduction pathway. Data represent mean ± standard deviation of FPKM values (n = 3). * significantly different from the control.

Gene name	Control	3.75‰	7.5‰
Auxin signaling transduction			
Auxin influx carrier (AUX1)	171.52 ± 15.44	226.57 ± 53.69	179.57 ± 64
Protein transport inhibitor response (TIR1)	79.51 ± 1.91	66.33 ± 1.63*	55.4 ± 7.59*
Auxin-responsive protein IAA (AUX/IAA)	758.43 ± 81.88	951.34 ± 242.18	841.73 ± 190.67
Auxin-response factor (ARF)	500.99 ± 35.17	503.3 ± 74.14	518.26 ± 73.38
Gretchen Hagen3 (GH3)	22.94 ± 4.50	30.51 ± 4.37	41.24 ± 4.28*
Small auxin-up RNAs (SAUR)	148.72 ± 6.16	372.31 ± 201.34*	198.64 ± 32.01*
Abscisic acid signaling transduction			
Abscisic acid receptor PYR/PYL	85.6 ± 8.15	84.79 ± 11.85	63.96 ± 6.80*
Protein phosphatase 2C (PP2C)	61.36 ± 5.72	54.94 ± 16.95	49.66 ± 16.78
Serine/threonine-protein kinase 2 (SNRK2)	125.34 ± 38.70	61.66 ± 3.64*	63.39 ± 12.11*
ABA responsive element binding factor (ABF)	115.52 ± 39.03	93.44 ± 7.26	140.67 ± 31.62
Ethylene signaling transduction			
Ethylene receptor (ETR)	117.91 ± 5.19	123.34 ± 10.71	116.55 ± 11.32
Serine/threonine-protein kinase CTR1 (CTR1)	76.04 ± 4.12	63.67 ± 1.47*	69.75 ± 2.00
Mitogen-activated protein kinase kinase (SIMKK)	145.32 ± 7.06	147.23 ± 16.06	189.52 ± 19.69*
Mitogen-activated protein kinase (MPK6)	271.28 ± 10.50	279.67 ± 35.74	279.25 ± 34.00
Ethylene-insensitive protein 2 (EIN2)	36.11 ± 4.02	33.53 ± 2.30	31.31 ± 2.86
Ethylene-insensitive protein 3 (EIN3)	116.19 ± 18.95	125.64 ± 20.36	115.86 ± 17.38
EIN3-binding F-box protein (EBF1/2)	173.16 ± 17.69	166.03 ± 15.49	128.30 ± 12.74*
Ethylene-responsive transcription factor (ERF1/2)	21.70 ± 8.16	63.20 ± 22.13*	58.11 ± 8.14*
Cytokinine signaling transduction			
Cytokinin receptor (CRE1)	123.27 ± 17.67	110.69 ± 3.4	117.30 ± 7.89
Histidine-containing phosphotransfer protein (AHP)	185.70 ± 36.97	196.76 ± 26.27	152.28 ± 18.46
Two-component response regulator ARR-B (B-ARR)	95.03 ± 1.39	107.12 ± 11.71	105.68 ± 5.63*
Two-component response regulator ORR (A-ARR)	67.03 ± 4.65	68.31 ± 12.48	86.75 ± 9.83*

Gibberellin signaling transduction			
Gibberellin receptor 1 (GID1)	183.10 ± 38.23	151.44 ± 12.50	162.61 ± 13.92
Gibberellin receptor 2 (GID2)	13.26 ± 0.49	20.60 ± 3.77	21.77 ± 5.36
Scarecrow-like protein (DELLA)	229.29 ± 29.98	184.62 ± 11.16	247.49 ± 35.27
Transcription factor (TF)	486.94 ± 32.76	447.5 ± 18.82	441.43 ± 65.28
Brassinosteroid signaling transduction			
Brassinosteroid insensitive 1 (BAK1)	34.01 ± 1.82	35.66 ± 3.90	29.17 ± 2.64*
Protein brassinosteroid insensitive 1 (BRI1)	11.3 ± 0.92	11.50 ± 0.42	14.37 ± 0.41*
BRI1 kinase inhibitor (BKI1)	52.43 ± 5.32	47.26 ± 12.97	42.52 ± 12.77
BR-signaling kinase (BSK)	23.41 ± 5.4	45.46 ± 11.26*	46.75 ± 27.16
Protein brassinosteroid insensitive 2 (BIN2)	107.12 ± 1.6	124.84 ± 17.53*	127.99 ± 20.92*
Brassinosteroid resistant 1/2 (BZR1/2)	32.12 ± 0.36	40.62 ± 4.18*	47.77 ± 14.31*
Xyloglucan:xyloglucosyl transferase (TCH4)	289.69 ± 103.35	947.5 ± 239.78*	891.90 ± 134.72*
Cyclin-D3 (CYCD3)	27.01 ± 4.77	36.67 ± 4.95*	32.26 ± 4.44
Jasmonic acid signaling transduction			
Jasmonic acid-amido synthetase (JAR1)	7.25 ± 3.35	6.93 ± 1.30	3.18 ± 1.68
Coronatine-insensitive protein 1 (COI1)	16.36 ± 1.19	18.67 ± 1.52	18.66 ± 2.11
Jasmonate ZIM domain-containing protein (JAZ)	451.36 ± 163.54	141.92 ± 36.39*	282.11 ± 114.32
Transcription factor MYC2 (MYC2)	83.50 ± 29.55	46 ± 17.48	61.32 ± 20.23
Salicylic acid signaling transduction			
Regulatory protein 1 (NPR1)	61.98 ± 8.94	56.95 ± 7.73	56.36 ± 7.27
Transcription factor (TGA)	21.50 ± 1.37	25.91 ± 5.78	29.16 ± 11.88
Pathogenesis-related protein 1 (PR1)	272.41 ± 36.8	283.18 ± 58.64	338.81 ± 53.18
